# Loop-Mediated Isothermal Amplification for Laboratory Confirmation of Buruli Ulcer Disease—Towards a Point-of-Care Test

**DOI:** 10.1371/journal.pntd.0004219

**Published:** 2015-11-13

**Authors:** Marcus Beissner, Richard Odame Phillips, Florian Battke, Malkin Bauer, Kossi Badziklou, Fred Stephen Sarfo, Issaka Maman, Agata Rhomberg, Ebekalisai Piten, Michael Frimpong, Kristina Lydia Huber, Dominik Symank, Moritz Jansson, Franz Xaver Wiedemann, Abiba Banla Kere, Karl-Heinz Herbinger, Thomas Löscher, Gisela Bretzel

**Affiliations:** 1 Department of Infectious Diseases and Tropical Medicine (DITM), University Hospital, Ludwig-Maximilians-University, Munich, Germany; 2 Komfo Anokye Teaching Hospital (KATH), Kwame Nkrumah University of Science and Technology (KNUST), Kumasi, Ghana; 3 Battke Scientia GmbH, Taufkirchen, Germany; 4 Institut National d’Hygiène (INH), Ministère de la Santé, Lomé, Togo; 5 Centre Hospitalier Régional Maritime (CHR-Maritime), Tsévié, Togo; 6 Kumasi Centre for Collaborative Research in Tropical Medicine (KCCR), Komfo Anokye Teaching Hospital (KATH), Kwame Nkrumah University of Science and Technology (KNUST), Kumasi, Ghana; 7 German Leprosy and Tuberculosis Relief Association, Togo office (DAHW-T), Lomé, Togo; University of Tennessee, UNITED STATES

## Abstract

**Background:**

As the major burden of Buruli ulcer disease (BUD) occurs in remote rural areas, development of point-of-care (POC) tests is considered a research priority to bring diagnostic services closer to the patients. Loop-mediated isothermal amplification (LAMP), a simple, robust and cost-effective technology, has been selected as a promising POC test candidate. Three BUD-specific LAMP assays are available to date, but various technical challenges still hamper decentralized application. To overcome the requirement of cold-chains for transport and storage of reagents, the aim of this study was to establish a dry-reagent-based LAMP assay (DRB-LAMP) employing lyophilized reagents.

**Methodology/Principal Findings:**

Following the design of an IS*2404* based conventional LAMP (cLAMP) assay suitable to apply lyophilized reagents, a lyophylization protocol for the DRB-LAMP format was developed. Clinical performance of cLAMP was validated through testing of 140 clinical samples from 91 suspected BUD cases by routine assays, i.e. IS*2404* dry-reagent-based (DRB) PCR, conventional IS*2404* PCR (cPCR), IS*2404* qPCR, compared to cLAMP. Whereas qPCR rendered an additional 10% of confirmed cases and samples respectively, case confirmation and positivity rates of DRB-PCR or cPCR (64.84% and 56.43%; 100% concordant results in both assays) and cLAMP (62.64% and 52.86%) were comparable and there was no significant difference between the sensitivity of the assays (DRB PCR and cPCR, 86.76%; cLAMP, 83.82%). Likewise, sensitivity of cLAMP (95.83%) and DRB-LAMP (91.67%) were comparable as determined on a set of 24 samples tested positive in all routine assays.

**Conclusions/Significance:**

Both LAMP formats constitute equivalent alternatives to conventional PCR techniques. Provided the envisaged availability of field friendly DNA extraction formats, both assays are suitable for decentralized laboratory confirmation of BUD, whereby DRB-LAMP scores with the additional advantage of not requiring cold-chains. As validation of the assays was conducted in a third-level laboratory environment, field based evaluation trials are necessary to determine the clinical performance at peripheral health care level.

## Author Summary

Buruli ulcer disease (BUD) mainly occurs in remote rural areas of Sub-Saharan Africa, affects skin and soft tissue, and may lead to severe disabilities. Therefore, early diagnosis and treatment with antimycobacterial therapy are essential whereby the WHO recommends laboratory confirmation of 70% of the cases. As the current diagnostic gold standard (polymerase chain reaction [PCR]) is restricted to third-level laboratories, development of confirmatory point-of-care (POC) tests for BUD applicable at primary health care level has become a research priority to bring diagnosis closer to where the patients are. Loop-mediated isothermal amplification (LAMP) has been selected by the WHO as one of the promising candidate technologies for POC tests. The aim of this study was to establish and validate a LAMP assay applying lyophilized reagents which are stable at ambient temperature, thus avoiding the need for cold-chains. The results from this study suggest that the assay provides a valuable alternative to other PCR tests as currently used for laboratory confirmation of BUD.

## Introduction

Buruli ulcer disease (BUD), caused by *Mycobacterium ulcerans*, is an infectious disease affecting skin, soft tissues and sometimes the bones. The major endemic foci occur in rural areas of Sub-Saharan Africa where BUD mainly affects children below the age of 15 years.

Antimycobacterial therapy can cure up to 80% of patients diagnosed in early stages of the disease. If treated in advanced stages or left untreated, extensive destruction of tissue followed by fibrous scarring and contractures may lead to severe sequelae such as functional limitation of affected joints, which occur in up to 25% of cases. In the absence of proven preventive strategies, early diagnosis and treatment are therefore crucial to avoid disease related disabilities [1–2].

The WHO recommends laboratory confirmation of at least 70% of clinically suspected BUD cases per country [3]. Application of the 100% *M*. *ulcerans* specific diagnostic reference standard for clinical samples, i.e. amplification of the multicopy insertion sequence (IS) *2404* by dry-reagent-based (DRB) PCR, conventional gel-based PCR (cPCR), or quantitative real-time PCR (qPCR) requires fully equipped molecular biology units with highly-skilled personnel and is thus mostly restricted to tertiary (reference) level laboratories or national research centres [4–9]. However, as the major burden of BUD exists in (remote) rural areas of endemic countries and up to one-third of BUD cases are diagnosed in advanced category III stages [10–12], molecular IS*2404* detection formats applicable as point-of-care (POC) tests are urgently needed to bring diagnosis closer to where the patients live [13].

Behind this background, an expert group convened by the Foundation for New Innovative Diagnostics (FIND) and the WHO in November 2013 selected loop-mediated isothermal amplification (LAMP) as promising nucleic acid based candidate POC technology applicable for decentralized diagnosis at primary health care level [14].

The salient features of LAMP technology are attributable to the *Bacillus stearothermophilus*-derived *Bst* polymerase, which is characterized by strand-displacement activity (without 5’-3’ exonuclease activity), enzyme activity at constant temperature (~ 65 +/- 3°C) without the need of steps for denaturation of double-stranded DNA or primer annealing at different temperatures, high amplification efficiency (up to 10^10^ copies in 60 minutes) and low susceptibility to classical PCR inhibitors (e.g. melanin, collagen, humic acids). Furthermore, the ability to specifically amplify target sequences by the use of four distinct primers recognizing 6 distinct regions in a single step without the need for sophisticated laboratory equipment made this nucleic acid detection method promising as POC test. LAMP applications were thus established and validated for the diagnosis of various human pathogens such as (protozoan) parasites (e.g. *Plasmodium falciparum*, *Leishmania spp*., *Trypanosoma brucei*, *Giardia duodenalis*, *Schistosoma mansoni*/*haematobium*, *Taenia solium*), bacteria (e.g. *Listeria monocytogenes*, *Staphylococcus aureus*, *Mycobacterium tuberculosis*) as well as viruses and fungi in settings with limited resources [15–25].

To date, three different LAMP assays for laboratory confirmation of BUD were published. The assay described by de Souza et al. targets the enoyl reductase gene of the *M*. *ulcerans* virulence plasmid, technical validation of the assay however was conducted only on a limited number of samples [26]. Njiru et al. and Ablordey et al. reported two LAMP assays amplifying different regions of the IS*2404* of *M*. *ulcerans*. Both assays underwent validation on various clinical and environmental samples of BUD patients and infected animals from Ghana and Australia, were 100% *M*. *ulcerans* specific (without any false positive result) and revealed analytical sensitivities of 20 [27] as well as 30–300 [28] copies of the respective IS*2404* target sequence, which equals 0.1 to 1.5 genome equivalents of *M*. *ulcerans*, respectively. These analytical sensitivities approach that of cPCR [6, 27–28], but not that of qPCR [7, 29]. However, both assays were evaluated under optimal laboratory conditions applying high-standard DNA extraction and purification procedures in third level laboratories or national research centers, which may not be practicable at primary health care level. To simulate technical feasibility under field conditions, crude (i.e. boiled) DNA extracts were used without further purification for LAMP testing of clinical samples and led to a significant decrease in sensitivity [28]. Moreover, all LAMP assays described so far require unlimited cold-chains as well as shipment of reagents on dry-ice, which is a major cost factor for endemic settings and not always feasible at decentralised facilities. Therefore, technical advancement of LAMP technology and DNA extraction into utterly field friendly formats is unanimously recommended [27–28].

Against this background, the aim of this study was to establish an IS*2404* detection based LAMP assay employing lyophilized reagents (dry-reagent-based [DRB] LAMP) which provides significant benefit for application under tropical climate conditions, to validate the assay on clinical samples including fine needle aspirates (FNA) which were largely omitted in previous studies, and to provide a prototype assay for future large-scale field testing.

## Materials and Methods

### Ethical statement

The study was approved by the Ghanaian KNUST (CHRPE/91/10) and the national Togolese (14/2010/CRBS) ethics committees. All samples analyzed in this study were collected for diagnostic purposes. Written informed consent was obtained from all study participants and/or their legal representative, if aged below 18 years.

### Study participants, clinical samples and data collection

Clinically suspected BUD patients were recruited from two study sites in Ghana (Agogo Presbyterian Hospital, Asante Akim North District, n = 12; Tepa Government Hospital, Ahafo Ano North District, n = 20) and one study site in Togo (“Centre Hospitalier Régional de Tsévié”, region “Maritime”, n = 59) and 140 diagnostic samples (FNA, n = 66; swab samples, n = 32; punch biopsy samples, n = 42) were collected according to standardized procedures. Briefly, swabs were taken by circling the undermined edges of ulcerative lesions, and FNA or 3mm punch biopsies were obtained from the center of non-ulcerative lesions. Samples were transported to the Kumasi Center for Collaborative Research in Tropical Medicine (KCCR, Kumasi, Ghana) or the “Institut National d’Hygiène” (INH, Lomé, Togo) in 2 ml screw cap tubes containing 700 μl (swab and punch biopsy samples) or 300 μl (FNA samples) cell lysis solution (CLS; Qiagen, Hilden, Germany) within one day at ambient temperature [10, 30–33].

Clinical, epidemiological and routine laboratory data were collected by means of WHO BU 01.N forms [34] and standardized project specific laboratory data entry forms, and were entered in a web-based database as previously described [10].

### Laboratory confirmation by PCR

Whole genome DNA was extracted from clinical samples in CLS at KCCR or INH using the Gentra Puregene DNA extraction kit (Qiagen) with minor modifications of the manufacturer’s instructions as described in S1 Protocol [6]. DNA extracts were stored at 4–8°C (up to one week) or -18°C (long-term storage).

For routine on-site laboratory confirmation DNA extracts were subjected to IS*2404* DRB PCR at KCCR and INH as previously described [6, 10, 35]. For comparative testing in the context of external quality assurance programs with conventional, gel-based IS*2404* PCR (cPCR) [4–5, 33, 35, 36] and a recently described modified IS*2404* qPCR based on the assay published by Fyfe et al. [7, 29] aliquots of DNA extracts were shipped to the Department of Infectious Diseases and Tropical Medicine (DITM), Munich, Germany by courier service at ambient temperature [10].

### Development of an IS*2404* detection-based LAMP assay

The development and validation of the LAMP assay was conducted in the laboratories of DITM.

#### DNA extracts

For establishment and technical validation of an IS*2404* LAMP assay, sequencing confirmed DNA extracts of five *M*. *ulcerans* strains from cultures (i.e. “must detect” samples), twelve mycobacterial species (i.e. “must not detect” samples: *M*. *avium*, *M*. *chelonae*, *M*. *fortuitum*, *M*. *gordonae*, *M*. *intracellulare*, *M*. *kansasii*, *M*. *marinum*, *M*. *smegmatis*, *M*. *szulgai*, *M*. *tuberculosis*, *M*. *xenopi* and *M*. *lentiflavum*) were available at DITM [29, 37–38].

#### IS*2404* plasmid standard

To generate a plasmid standard applicable as positive control and calibration template with known copy numbers for regions amplified by gel-based IS*2404* (DRB) PCR, IS*2404* qPCR as well as the novel IS*2404* LAMP assays, respectively, the complete IS*2404* sequence was amplified by conventional PCR from a sequencing confirmed *M*. *ulcerans* culture extract. The primers were IS*2404*-fwd (5`-3`: ATG GCT TTG TTG GCG ATC GC) and IS*2404*-rev (5`-3`: TTA GCA GGC TTG TGA GCT GG). The reaction mixture contained 13.5 μl molecular grade H_2_O (Carl Roth, Karlsruhe, Germany), 2.5 μl 10-fold PCR buffer for *Thermococcus kodakaraenis* (KOD) derived DNA polymerase (Merck, Darmstadt, Germany), 2.0 μl MgSO_4_, 2.5 μl dNTP mix (2 mM each), 10 pmol of each primer, 2 μl DNA template and 0.5 μl KOD Hot-Start Polymerase (Merck). The amplification was performed at 95°C for 2 min., followed by 35 cycles of 95°C for 20 sec., 57°C for 15 sec., 70°C for 20 sec. and a final incubation at 70°C for 2 min. The PCR product was purified from a 1.2% agarose TAE gel by means of the Double Pure Kit (Bio&SELL, Feucht, Germany) according to the manufacturer’s instructions. Then, a 3’A-overhang was added to the purified PCR product by incubating the following reaction mixture for 20 minutes at 72°C: 38 μl purified PCR product, 5 μl PCR-reaction buffer (10-fold), 5 μl MgCl_2_ (25 mM), 1 μl dATP (10 mM), 1 μl Taq polymerase (Bio&SELL, each). The ‘A’-tailed PCR product was again purified with the Double Pure Kit and then ligated into a pGEM-T-vector (Promega, Mannheim, Germany) using the following reaction mixture incubated at 4°C overnight: 5 μl 2-fold Rapid Ligation Buffer, 3 μl DNA (with 3’A-overhangs), 1 μl pGEM-T-vector and 1 μl T4 DNA Ligase. For cloning the plasmid to bacteria, 2 μl of the ligation reaction were mixed with 50 μl *E*. *coli* JM109 z-competent cells (Zymo, Freiburg, Germany), inoculated onto LB agar plates with 100 mg/L ampicillin (Carl Roth) and incubated at 37°C overnight. The selected *E*. *coli* clone was cultivated in 5 ml LB medium with 100 mg/L ampicillin (Carl Roth), incubated at 37°C overnight and then subjected to plasmid preparation using the matrix-based HiYield Plasmid Mini kit (Süd-Laborbedarf, Gauting, Germany) according to the manufacturer’s specification. The cloned plasmid sequence was confirmed by direct DNA sequencing as previously described [37]. Purity of extracted IS*2404* plasmids was assessed by photometry on a BioPhotometer plus (Eppendorf, Wesseling-Berzdorf, Germany) and agarose gel-electrophoresis on a 1% TAE gel. Quantification of plasmid DNA extracts was done using the fluorescence quantification kit “Quant-It dsDNA Broad Range” on a Qubit (Life Technologies, Karlsruhe, Germany) according to the manufacturer’s instruction and plasmid standards were adjusted to 10^6^ copies per μl.

Analytical sensitivity was determined as lower limit of detection (LOD), i.e. lowest template concentration rendering positive amplification of 95% of samples. The LOD of each assay (including DRB PCR, cPCR and qPCR) was determined at DITM using the plasmid standard in 10-fold serial dilutions [39].

#### IS*2404* LAMP primers

**Fig 1:**
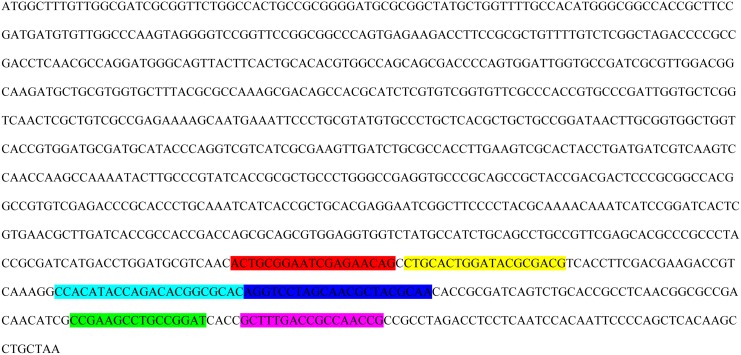
Binding sites of LAMP primers within the IS*2404*. Fig 1 shows binding sites of LAMP primers within the IS*2404* of *M*. *ulcerans* strain Agy 99 (Genbank accession number: CP000325.1). Primer MU2-FIP consists of a first reverse complementary region “F1” and a second forward region “F2”. Primer MU2-BIP (first described by Ablordey et al. [28]) consists of a first forward region “B1” and a second reverse complementary region “B2”. Primers and corresponding regions are highlighted in colors; red: primer MU2-F3 (region “F3”); yellow: primer MU2-FIP (region “F2”); light blue: primer MU2-FIP (region “F1”); dark blue: primer MU2-BIP (region “B1”); green: primer MU2-BIP (region “B2”) and pink: primer MU2-B3 (region “B3”; first described by Ablordey et al. [28]).

A set of four primers was designed for amplification of the *M*. *ulcerans* specific IS*2404* by manually analyzing the target sequence and designing the primers according to the needs for LAMP amplification. Specificity of the primers for *M*. *ulcerans* was confirmed in silico by means of the basic local alignment search tool (BLAST, GenBank, NCBI) [40]. The primer set consisted of two smaller oligonucleotides with forward (MU2-F3) and reverse complementary (MU2-B3) sequences and two larger oligonucleotides (MU2-FIP and MU2-BIP) with a complex sequence construction. Primer sequences are provided in Table 1 and binding sites within the IS*2404* are displayed in Fig 1. Primers MU2-B3 and MU2-BIP were first published by Ablordey et al. [28].

**Table 1:** Primer sequences of the IS*2404* LAMP assay.

Primer	Sequence (forward)	Sequence (reverse complementary)	Complete sequence^a^
MU2-F3	ACT GCG GAA TCG AGA ACA G	N/A	ACT GCG GAA TCG AGA ACA G
MU2-B3^b^	N/A	CGG TTG GCG GTC AAA GC	*CGG TTG GCG GTC AAA GC*
MU2-FIP		GTG CGC CGT GTC TGG TAT GTG G	*GTG CGC CGT GTC TGG TAT GTG G*CT GCA CTG GAT ACG CGA CG
	CTG CAC TGG ATA CGC GAC G		
MU2-BIP^c^	AGG TCC TAG CAA CGC TAC GCA		AGG TCC TAG CAA CGC TAC GCA *AAT CCG GCA GGC TTC GG*
		AAT CCG GCA GGC TTC GG	

Table 1 shows the sequences of a set of four *M*. *ulcerans* specific LAMP primers targeting the IS*2404*.

N/A, not applicable.

^a^ The reverse complementary sequence of nucleotides is displayed in italics.

^b^ MU2-B3, the same primer sequence was originally published by Ablordey et al. as primer “Buruli-B3” [28].

^c^ MU2-BIP, the same primer sequence was originally published by Ablordey et al. as primer “Buruli-BIP” [28].

#### Conventional IS*2404* LAMP (cLAMP) protocol

Each cLAMP reaction mix consisted of 1 μl *Bst* DNA polymerase (large fragment, 8 U/μl; New England Biolabs [NEB], Frankfurt am Main, Germany), 1.0 μl dNTP mix (2 mM each, Merck), 1.0 μl of primers MU2-F3 and MU2-B3 (5 pmol/μl) and 2.0 μl of primers MU2-FIP and MU2-BIP (10 pmol/μl), respectively (TibMolBiol, Berlin, Germany), 2.0 μl betaine (5 M; Sigma-Aldrich, Taufkirchen, Germany), 2.5 μl 10-fold Thermopol buffer for *Bst* DNA polymerase (NEB) and 11.5 μl molecular grade H_2_O (Carl Roth). Following the addition of 1 μl DNA extract (template), cLAMP reactions (final volume: 25 μl) were carried out in 1.5 ml SafeSeal reaction tubes (Sarstedt, Nümbrecht, Germany) at 65°C for 60 minutes in a conventional thermoblock (HLC Thermomixer MKR 13, HLC BioTech, Bovenden, Germany) and a final step at 80°C for 10 minutes terminated the amplification.

Each run included negative extraction and no template (H_2_O) as well as positive controls.

#### IS*2404* dry-reagent-based LAMP (DRB LAMP) protocol

The DRB LAMP reaction mix contained the same concentrations of reagents and primers as described for the conventional LAMP assay. However, as the wildtype *Bst* polymerase large fragment contained glycerol it was not possible to lyophilize. Therefore, a customized *Bst* polymerase dissolved in H_2_O and reaction buffer (NEB) were applied for the DRB LAMP assay. The DRB LAMP reaction mix was prepared for each reaction in 1.5 ml reaction tubes and subjected to lyophilization by means of a RVC 2–25 CD plus Vacuum Concentrator (Christ, Osterode, Germany) at 1.0 mbar and a safety pressure of 1.000 mbar according to the manufacturer’s specifications. During the process of validation, DRB LAMP reaction tubes were stored at ambient temperature in the dark and reactions were carried out within one week after lyophilization as described for cLAMP following the addition of 1 μl template DNA and adjustment with 24 μl molecular grade H_2_O (Carl Roth) to a final reaction volume of 25 μl.

#### LAMP product visualization

LAMP products were detected by two different methods: i) gel-electrophoresis on a 0.5x TBE gel containing 0.01% GelRed (Biotium, Cologne, Germany) and ii) SYBR Green I (Life Technologies) staining (0.5 μl of 1:5 diluted SYBR Green I staining solution and 12.5 μl of LAMP product) followed by UV-transillumination.

### Comparative testing of clinical samples

#### Confirmation rates of routine assays (PCR [DRB PCR, cPCR] and qPCR) compared with cLAMP

The confirmation rate was defined as the number of patients with a positive PCR, qPCR or cLAMP test result divided by the number of all suspected BUD cases.

#### Sensitivity rates of PCR (DRB PCR, cPCR) and cLAMP among confirmed BUD patients

The sensitivity rate was defined as the number of patients with a positive PCR or cLAMP test result divided by the number of all qPCR confirmed patients.

#### Positivity rates of routine assays (PCR [DRB PCR, cPCR] and qPCR) and cLAMP among clinical samples from suspected BUD cases

The positivity rate was defined as the number of clinical samples from suspected BUD cases with a positive PCR, qPCR or cLAMP test result divided by the number of all samples tested.

#### Sensitivity rates of routine assays (PCR [DRB PCR, cPCR]) compared with cLAMP among clinical samples from confirmed BUD cases

The sensitivity rate was defined as the number of clinical samples from confirmed BUD patients with a positive PCR or cLAMP test result divided by the number of all samples with a positive qPCR result.

#### Positivity rates of DRB LAMP compared with routine assays (PCR [DRB PCR, cPCR] and qPCR) and cLAMP among clinical samples from suspected BUD cases

The positivity rate was defined as the number of clinical samples with a positive PCR, qPCR or cLAMP test result divided by the number of all samples tested.

#### Sensitivity rates of cLAMP and DRB LAMP among clinical samples from confirmed BUD cases

The sensitivity rate was defined as the number of clinical samples from confirmed BUD patients with a positive cLAMP or DRB LAMP test result divided by the number of all samples with a positive result in DRB PCR, cPCR and qPCR each.

### Statistical analysis

The study was observational and transversal (cross-sectional study design). An approximative test (McNemar chi-square test for matched pairs of samples with categorical test results) and estimation of standard error of proportion (to calculate 95 percent confidence intervals [95%-CI] of categorical test results) were conducted. Significant differences were defined as *P*-values below 0.05 or as not overlapping of 95%-CI of proportions.

## Results

### Performance characteristics of routine assays (DRB PCR, cPCR and qPCR) compared with cLAMP

In silico analysis of the novel IS*2404* LAMP primers and testing of 17 DNA extracts from mycobacterial cultures (*M*. *ulcerans*, n = 5; other mycobacteria, n = 12) by cLAMP and DRB LAMP revealed 100% specificity of both assays for *M*. *ulcerans*. The LODs were 50 (DRB PCR and cPCR), 3 (qPCR) and 100 (cLAMP) copies of the target sequence IS*2404*, corresponding to 0.2, 0.01, and 0.5 *M*. *ulcerans* genome equivalents, respectively.

### Laboratory confirmation of suspected BUD cases

Out of the 91 patients with suspected BUD, 68 were laboratory confirmed as BUD patients (74.73%) by routine methods. Among 68 confirmed BUD patients, 40 patients (58.82%) were in age group 5–14 years (age range 5–56 years, mean 14 years, median 11 years), 33 patients (48.53%) were male, and 36 patients (52.94%) presented with non-ulcerative lesions.

### Confirmation rates of routine assays (DRB PCR, cPCR and qPCR) compared with cLAMP among suspected BUD cases

DRB PCR (on-site at KCCR or INH) and cPCR (DITM, 100% concordance between DRB and cPCR results) confirmed 59/91 (64.84%; 95%-CI: 55.02%-74.65%) of the suspected BUD cases, the qPCR confirmed 68/91 (74.73%; 95%-CI: 65.80%-83.65%), thus added an additional diagnostic yield of 9.89%. The confirmation rate for cLAMP was 62.64% (95%-CI: 52.70%-72.58%; n = 57). Neither DRB PCR nor cPCR or cLAMP had false positive results compared with qPCR, and confirmation rates were not significantly different. According to McNemar test, there was no significant difference between DRB PCR or cPCR (100% concordant results) compared with qPCR (OR_crude_ = 1.60; 95%-CI: 0.81–3.20; *P*-value = 0.15), between cLAMP compared with qPCR (OR_crude_ = 1.76; 95%-CI: 0.89–3.50; *P*-value = 0.08) and between DRB PCR or cPCR compared with cLAMP (OR_crude_ = 1.10; 95%-CI: 0.57–2.11; *P*-value = 0.76).

### Sensitivity rates of DRB PCR, cPCR and cLAMP among confirmed BUD cases

Among the 68 BUD cases confirmed by qPCR, the sensitivity was 86.76% (95%-CI: 78.71%-94.82%; n = 59) for DRB PCR and cPCR and 83.82% (95%-CI: 75.07%-92.58%; n = 57) for cLAMP. According to McNemar test, there was no significant difference between DRB PCR and cPCR compared with cLAMP (OR_crude_ = 1.27; 95%-CI: 0.44–3.63; *P*-value = 0.63).

### Positivity rates of routine assays (DRB PCR, cPCR and qPCR) and cLAMP among clinical samples from suspected BUD cases

Among the 140 samples from 91 clinically suspected BUD cases, the positivity rate was 56.43% (95%-CI: 48.21%-64.64%; n = 79) for DRB PCR and cPCR, 67.14% (95%-CI: 59.36%-74.92%; n = 94) for qPCR, and 52.86% (95%-CI: 44.59%-61.13%; n = 74) for cLAMP. Neither DRB PCR nor cPCR or cLAMP revealed false positive results compared with qPCR. According to McNemar test, there was no significant difference between DRB PCR or cPCR compared with qPCR (OR_crude_ = 1.58; 95%-CI: 0.94–2.64; *P*-value = 0.07) and between DRB PCR or cPCR compared with cLAMP (OR_crude_ = 1.16; 95%-CI: 0.70–1.90; *P*-value = 0.55), whereas the difference between cLAMP compared with qPCR was significant (OR_crude_ = 1.82; 95%-CI: 1.09–3.05; *P*-value = 0.02).

Stratification into sample types did not reveal significant differences in positivity rates of DRB PCR, cPCR, qPCR and cLAMP among FNA, swab or punch biopsy samples.

### Sensitivity rates of routine assays (DRB PCR and cPCR) compared with cLAMP among clinical samples from confirmed BUD cases

Among the 94 samples from 68 BUD cases confirmed by qPCR, the sensitivity was 84.04% (95%-CI: 76.64%-91.45%; n = 79) for DRB PCR and cPCR, and 78.72% (95%-CI: 44.59%-61.13%; n = 74) for cLAMP. According to McNemar test, there was no significant difference between DRB PCR or cPCR compared with cLAMP (OR_crude_ = 1.42; 95%-CI: 0.64–3.18); *P*-value = 0.35).

### Performance characteristics of DRB LAMP

DRB LAMP revealed the same performance characteristics as determined for cLAMP (i.e. 100% *M*. *ulcerans* specificity and a LOD of 0.5 *M*. *ulcerans* genome equivalents).

### Positivity rates of DRB LAMP compared with routine assays (DRB PCR, cPCR and qPCR) and cLAMP among clinical samples from suspected BUD cases

To compare DRB LAMP with DRB PCR, cPCR, qPCR and cLAMP, 32 samples (FNA and swab samples, n = 16, respectively) from 32 suspected BUD cases were subjected to the assays. The positivity rate was 75.0% (95%-CI: 62.41%-87.59%; n = 24) for DRB PCR, cPCR and qPCR, 71.88% (95%-CI: 87.84%-100%; n = 23) for cLAMP, and 68.75% (95%-CI: 80.61%-100%; n = 22) for DRB LAMP. Neither cLAMP nor DRB LAMP revealed false positive results compared with DRB PCR, cPCR and qPCR.

According to McNemar test, there was no significant difference neither between DRB PCR, cPCR or qPCR compared with cLAMP (OR_crude_ = 1.17; 95%-CI: 0.34–4.10; *P*-value = 0.78), nor between DRB PCR, cPCR or qPCR compared with DRB LAMP (OR_crude_ = 1.36; 95%-CI: 0.40–4.49; *P*-value = 0.58), nor between cLAMP compared with DRB LAMP (OR_crude_ = 0.86; 95%-CI: 0.26–2.87; *P*-value = 0.79).

### Sensitivity rates of cLAMP and DRB LAMP among clinical samples from confirmed BUD cases

Among the 24 samples from 24 BUD patients confirmed by DRB PCR, cPCR and qPCR, the sensitivity was 95.83% (95%-CI: 87.84%-100%; n = 23) for cLAMP and 91.67% (95%-CI: 80.61%-100%; n = 22) for DRB LAMP. According to McNemar test, there was no significant difference between cLAMP compared with DRB LAMP (OR_crude_ = 0.48; 95%-CI: 0.02–7.54); *P*-value = 0.56).

### IS*2404* LAMP detection methods

Out of 74 amplicons derived from cLAMP reactions, 74/74 (100%) were judged positive by gel-electrophoresis and 73/74 (98.65%) by SYBR Green I staining. All products derived from DRB LAMP were likewise analyzed and the concordance rate was 22/22 (100%) between both detection methods.

Table 2 shows confirmation rates, sensitivity, specificity and significance of the applied molecular tests.

**Table 2:** Confirmation rates, sensitivity, specificity and significance of the applied molecular tests.

Number of BUD cases	Number of samples^a^	Statistical parameter	DRB PCR and cPCR^b^	qPCR	cLAMP	DRB LAMP
91 suspected	140	Positive results	59	68	57	N.A.
		Confirmation rate [%]	64.84	74.73	62.64	N.A.
		95%-CI^c^ [%]	55.02–74.65	65.80–83.65	52.70–72.58	N.A.
		Specificity^d^ [%]	100	N.A.	100	N.A.
68 confirmed^e^	140	Positive results	59	68	57	N.A.
		Sensitivity [%]	86.76	N.A.	83.82	N.A.
		95%-CI^c^ [%]	78.71–94.82	N.A.	75.07–92.58	N.A.
91 suspected	140^#^	Positive results	79	94	74	N.A.
		Positivity rate [%]	56.43	67.14*	52.86*	N.A.
		95%-CI^c^ [%]	48.21–64.64	59.36–74.92	44.59–61.13	N.A.
		Specificity^d^ [%]	100	N.A.	100	N.A.
68 confirmed^e^	94^#^	Positive results	79	94	74	N.A.
		Sensitivity [%]	84.04	100	78.72	N.A.
		95%-CI^c^ [%]	76.64–91.45	N.A.	44.59–61.13	N.A.
32 suspected	32	Positive results	24	24	23	22
		Positivity rate [%]	75.00	75.00	71.88	68.75
		95%-CI^c^ [%]	62.41–87.59	62.41–87.59	87.84–100	80.61–100
		Specificity^d^ [%]	100	N.A.	100	100
24 confirmed^e^	24	Positive results	N.A.	N.A.	23	22
		Sensitivity [%]	N.A.	N.A.	95.83	91.67
		95%-CI^c^ [%]	N.A.	N.A.	87.84–100	80.61–100
		Specificity^d^ [%]	N.A.	N.A.	100	100

Table 2 shows the results of DRB PCR, cPCR, qPCR, cLAMP, and DRB LAMP from clinical samples of clinically suspected and confirmed BUD cases recruited at Agogo Presbyterian Hospital, Ghana, Tepa Government Hospital, Ghana, and at Centre Hospitalier Régional de Tsévié, Togo.

N.A., not applicable.

^a^ Number of clinical samples tested.

^#^ indicates if the presented results refer to the number of samples–all other results refer to the number of patients or ^¶^the number of patients and samples was equal.

^b^ Results of the DRB PCR and cPCR were 100% concordant for all samples tested.

^c^ 95 percent confidence interval.

^d^ Specificity was calculated as proportion of truly positive test results out of all positive results of the same test, based on the results of qPCR as reference test.

^e^ Laboratory confirmation was defined as positive IS*2404* qPCR test result of any sample tested per patient.

* Significantly different proportions of positive results among all clinical samples tested by two different tests, calculated by McNemar test.

## Discussion

BUD belongs to the currently five neglected diseases in line for the IDM (innovative and intensified disease management) approach demanding a major scaling up of active detection, treatment, monitoring and surveillance. Development of diagnostic tests that bring health services closer to where NTDs are is considered a research priority. LAMP, a technology that features cost effectiveness, robustness and modest needs in terms of equipment, has recently been selected by the WHO as one of the promising tools for decentralized diagnostics [13–14, 41].

Several investigators recently succeeded in developing *M*. *ulcerans* specific LAMP assays which showed performance characteristics comparable to conventional PCR formats [26–28]. Based on longstanding experience with a DRB PCR format for laboratory confirmation of BUD in Ghana and Togo [6, 10, 30, 33, 36, 42], the development of a DRB LAMP assay applicable under tropical climate conditions at primary health care level was envisaged in this study. Thermodynamic reasons (i.e. leaving out an initial denaturation step for annealing of primers) required the design of modified primers, therefore as a first step a new cLAMP assay was established that constituted the basis for the DRB format. During development of the DRB assay lyophilization of the reaction mix initially constituted a major challenge. Due to the glycerol content of *Bst* polymerase and reaction buffer as employed in previous cLAMP formats (including our own), customized glycerol-free reagents had to be obtained and adequate lyophilization protocols had to be established.

The comparable performance of cLAMP, DRB LAMP and DRB PCR as well as cPCR suggests that both LAMP formats constitute a reliable alternative to conventional routine assays. Our data also show that the LAMP assays and the DRB PCR as well as cPCR have equal sensitivity for FNA samples. Both LAMP formats are applicable at primary health care level, the DRB format however provides significant advantages such as a simplified test layout and the possibility of storage of reagents at ambient temperature. Decentralized utilization of LAMP technology furthermore would lead to cost saving due to reduced expenditures for transportation of samples to a reference center as well as reduced test costs, i.e. US$ 1–2 per LAMP reaction as compared to US$ 8–10 per DRB PCR or cPCR reaction.

In this study it was not possible to assess long-term storage of DRB-LAMP reaction tubes under tropical conditions. Long-term storability of DRB PCR reaction tubes was however previously proven [6, 36] which allows the conclusion that maximum storage periods of up to 12 months also apply for LAMP reagents.

Although in our study routine PCR and LAMP assays for the most part did not perform significantly different from qPCR, it must be assumed that qPCR renders an additional diagnostic yield of approximately 10% [10]. Therefore, regardless of the method used, confirmation of negative samples by qPCR e.g. through the global network of laboratories for confirming *M*. *ulcerans* infection [43] should be attempted. Likewise, participation of laboratories in external quality assurance programs as implemented by Eddyani et al. in collaboration with the WHO is strongly recommended [44].

While the amplification procedure of LAMP technology especially in the DRB format can be considered field friendly without restriction, current DNA extraction procedures are not yet entirely appropriate for POC testing and need optimization. As shown by Ablordey et al. the use of boiled crude DNA extracts led to a significant decrease in sensitivity [28]. Other options such as one-tube silica-membrane based extraction protocols [45] or one-tube enzyme-based lyophilized reactions are yet to be evaluated. A field friendly approach to storage of DNA extracts for purposes of quality assurance could be the filter paper technology as successfully applied for TBC [46].

In conclusion, the cLAMP and DRB LAMP formats evaluated in this study are equivalent alternatives to conventional PCR techniques and, provided the availability of field friendly DNA extraction formats, constitute valuable tools for decentralized laboratory confirmation of BUD. As in the case of other investigators who previously developed BUD specific LAMP assays, the validation of the LAMP assays presented in this study was conducted in a third-level laboratory environment, therefore field based evaluation trials are necessary to determine the clinical performance at peripheral health care level.

## Supporting Information

S1 Protocol Extraction of mycobacterial DNA from clinical specimens. (PDF)
